# Contrast-enhanced ultrasound to predict malignant upgrading of atypical ductal hyperplasia

**DOI:** 10.1186/s13058-024-01772-2

**Published:** 2024-02-12

**Authors:** Jun Kang Li, Zhi Ying Jin, Yong Jie Xu, Nai Qin Fu, Ying Jiang, Shi Yu Li, Rui Lan Niu, Gang Liu, Zhi Li Wang

**Affiliations:** 1 Department of Ultrasound, The First Medical Center, Chinese PLA General Hospital, 28 Fuxing Road, Beijing, 100853 China; 2 Department of Ultrasound, Chinese PLA 63820 Hospital, Mianyang, Sichuan China; 3 Department of Ultrasound Diagnosis, Strategic Support Force Medical Center of Chinese PLA, Beijing, China; 4 Department of Radiology, The First Medical Center, Chinese PLA General Hospital, Beijing, China

**Keywords:** Atypical ductal hyperplasia, Ultrasound, Contrast media, Biopsy

## Abstract

**Background:**

A malignancy might be found at surgery in cases of atypical ductal hyperplasia (ADH) diagnosed via US-guided core needle biopsy (CNB). The objective of this study was to investigate the diagnostic performance of contrast-enhanced ultrasound (CEUS) in predicting ADH diagnosed by US-guided CNB that was upgraded to malignancy after surgery.

**Methods:**

In this retrospective study, 110 CNB-diagnosed ADH lesions in 109 consecutive women who underwent US, CEUS, and surgery between June 2018 and June 2023 were included. CEUS was incorporated into US BI-RADS and yielded a CEUS-adjusted BI-RADS. The diagnostic performance of US BI-RADS and CEUS-adjusted BI-RADS for ADH were analyzed and compared.

**Results:**

The mean age of the 109 women was 49.7 years ± 11.6 (SD). The upgrade rate of ADH at CNB was 48.2% (53 of 110). The sensitivity, specificity, positive predictive value, and negative predictive value of CEUS for identification of malignant upgrading were 96.2%, 66.7%,72.9%, and 95.0%, respectively, based on BI-RADS category 4B threshold. The two false-negative cases were low-grade ductal carcinoma in situ. Compared with the US, CEUS-adjusted BI-RADS had better specificity for lesions smaller than 2 cm (76.7% vs. 96.7%, *P* = 0.031). After CEUS, 16 (10 malignant and 6 nonmalignant) of the 45 original US BI-RADS category 4A lesions were up-classified to BI-RADS 4B, and 3 (1 malignant and 2 nonmalignant) of the 41 original US BI-RADS category 4B lesions were down-classified to BI-RADS 4A.

**Conclusions:**

CEUS is helpful in predicting malignant upgrading of ADH, especially for lesions smaller than 2 cm and those classified as BI-RADS 4A and 4B on ultrasound.

## Background

Atypical ductal hyperplasia (ADH) is a clonal proliferative lesion with pathological features resembling those of low-grade ductal carcinoma in situ (DCIS) [1]. Lesions with a contiguous extent of less than or equal to 2 mm or with less than 2 involved duct spaces are classified as ADH based on size/extent criteria; otherwise, they should be classified as DCIS [1]. However, the criteria were established based on excisional biopsy results. The size/extent of the tumor is difficult to accurately determine through percutaneous biopsy specimens, particularly in core needle biopsies (CNB) where the entire lesion may not be visible [1, 2]. ADH is currently diagnosed in 3% to 4% of breast CNB guided by imaging [3]. A systematic review revealed that 42% of ADH lesions diagnosed via US-guided CNB were upgraded to malignancy after surgery, and suggested that all cases of ADH diagnosed with needle biopsy should undergo surgical excision [4]. However, a recent study demonstrated that observation may also be safe for selected ADH patients, with only 4.4% of patients diagnosed with cancer during a median follow-up period of 5.2 years [5]. Overtreatment and undertreatment can present a dilemma for physicians when making clinical decisions for patients with ADH. Accurately predicting which ADH lesions are likely to upgrade to malignancy presents a significant challenge in implementing individualized risk management.

Previous studies have attempted to use imaging and pathology methods to identify ADH underestimated by CNB to reduce unnecessary open excision [6–13]. Factors such as age, family history, calcifications on mammography or ultrasound, suspicious enhancement on MRI, discordance between pathology and imaging results, and extent or number of ADH foci on histopathology were previously associated with malignancy upgrading [2, 10–13]. However, these factors are inadequate for identifying the upgrade and non-upgrade of ADH in the clinical setting. Contrast-enhanced ultrasound (CEUS) and optoacoustic imaging can detect microvascular within target lesions, improving the diagnostic accuracy of ultrasound. Optoacoustic imaging can detect hemoglobin to distinguish hypoxic and normal oxygenated tissue [14]. However, not all lack of hemoglobin signals necessarily indicate hypoperfusion. Tissue with blood volume below the detection threshold does not show any blood signal on optoacoustic imaging. CEUS can help to distinguish the poorly perfused regions and non-perfusion areas of the tumor that cannot be distinguished by optoacoustic imaging [15]. Studies have shown that CEUS performs exceptionally well in distinguishing between benign and malignant breast and thoracic wall lesions [16, 17]. However, no studies have been performed to evaluate the value of CEUS in ADH.

Thus, the purpose of this study was to investigate the diagnostic performance of CEUS in predicting ADH diagnosed by US-guided CNB that was upgraded to malignancy after surgery.

## Methods

### Study population

The retrospective study was approved by the institutional review board of our hospital and informed consent was waived because of the retrospective design. The database was searched to identify the patients who were diagnosed with ADH via US-guided CNB at our institution from June 2018 to June 2023. A total of 167 consecutive women were identified. Because CEUS was not a routine diagnostic procedure for breast at our institution, it was only performed on patients who had been recommended to undergo CEUS following a multidisciplinary meeting for presurgical evaluation. Recommendations for CEUS are mainly based on family history of breast cancer, physician's experience, and patient's preference for non-open excision. Out of the 167 women, 49 were excluded due to the absence of a CEUS examination, leaving 118 patients who underwent CEUS and were initially included in this study. Exclusion criteria for this study included patients with a history of malignant breast disease (*n* = 1), those with additional malignant lesions in the ipsilateral or contralateral breast at the time of diagnosis (*n* = 3), those confirmed to have lymph node metastasis preoperatively (*n* = 1), and those who did not underwent surgery (*n* = 4). Ultimately, 109 women with 110 lesions were included in the study, with one patient presenting with ADH lesions in both breasts. All 109 women underwent surgery, and the reference standard for the study was the pathologic results of the surgeries.

### US and CEUS examination procedures

All breast US and CEUS examinations were performed by two radiologists (J.Y., W.Z.L., with 7 and 20 years of experience in breast imaging) using an ACUSON S2000 ultrasound system (Siemens, Berlin, Germany) with a 9L4 linear array probe. The images of lesions were stored. The size (the maximum diameter) and location (clock-face location, distance from the nipple, and distance from the skin to the anterior aspect of the lesions) of lesions were recorded. Then, the plane with the maximum diameter or rich color Doppler flow signal, as well as with appropriate surrounding tissues was selected as standard plane for CEUS. This plane remained unchanged throughout the procedure, with the display set to dual-image mode and the mechanical index adjusted to 0.06–0.08. The contrast agent was SonoVue (Bracco Suisse SA, Italy), which consisted of 59 mg of sulfur hexafluoride microbubbles, prepared by mixing 5 ml of saline with dry powder and shaking to generate a suspension. A 20-gauge cannula was inserted through the antecubital vein to administer a bolus of 5.0 ml of the contrast agent, followed by an infusion of 5–10 ml of saline. Real-time images of CEUS were recorded for at least 3 min.

### US-guided CNB procedures

Breast biopsies were conducted with US guidance by two experienced radiologists (W.Z.L. and L.J.K.), with 20 and 10 years of experience respectively, using a MyLab Twice US system (Esaote, Italy) with a LA523 linear array probe, and a 16- or 18-gauge core biopsy needle (Bard Peripheral Vascular, Inc, USA). Multiple samples were taken from various areas within the targeted lesions. Penetration depths were set at 22 mm, and 2–5 samples were collected from each targeted lesion. The number of samplings performed on each lesion was determined by the quality of the specimen. If the sample is complete, the biopsy procedure is terminated after 2–3 repeated samplings. However, if the sample is fragmented, additional samplings are required. The location, needle gauge, and number of cores were recorded.

### Image analysis

Image analysis were independently performed by two radiologists (J.Y. and Z.Y.J., with more than 5 years of experience in breast US) who were blinded to the initial imaging reports and pathology and finally surgical pathology results. Disagreements were adjudicated and resolved by a third radiologist (W.Z.L.). US features were analyzed according to the American College of Radiology Breast Imaging Reporting and Data System (BI-RADS) [18]. Lesions were categorized as 3, 4A, 4B, 4C, or 5.

CEUS features were analyzed based on literature review [19, 20] and were described as follows: enhancement degree: non-enhancement, hypo-enhancement, iso-enhancement, and hyper-enhancement (reference to surrounding tissue); enhancement direction: centripetal, centrifugal, or diffuse; internal homogeneity: homogeneous or heterogeneous; enhancement shape: round, oval, and irregular; enhancement margin: well-defined, ill-defined; perfusion defects; size on CEUS: enlarged or not; radial or penetrating vessels; wash-in time: earlier, meantime, and late. Then, all lesions were scored on a scale of 1–5 points according to the scoring system reported by Xiao et al. [19]. One point: no enhancement inside the lesion; 2 points: iso-enhancement or hypo-enhancement compared with the surrounding tissue; 3 points: earlier wash-in time, homogeneous or heterogeneous, well-defined enhancement margin, round or oval enhancement shape, and not enlarged size on CEUS; 4 points: earlier wash-in time, heterogeneous, enlarged size on CEUS, without perfusion defects and radial or penetrating vessels; 5 points: heterogeneously enhanced, with enlarged size on CEUS, with or without perfusion defects, usually with radial or penetrating vessels. Finally, CEUS was incorporated into US BI-RADS yielded a CEUS-adjusted BI-RADS according to the following protocol: if the lesion scored 1–2 points, the US BI-RADS category would be reduced by one level (US BI-RADS category 3 remained unchanged); if the lesion scored 3 points, the US BI-RADS category remained unchanged; if the lesion scored 4–5 points, the US BI-RADS category would be increased by one level (US BI-RADS category 5 remained unchanged).

### Statistical analysis

A malignancy that was found at surgery in cases of ADH diagnosed via US-guided CNB was defined as upgrade. The distribution of continuous data was evaluated by using the Shapiro–Wilk test. Normally distributed continuous data were expressed as means ± standard deviations and were compared by using Student’s t-tests. Non-normally distributed continuous data were expressed as medians and interquartile ranges and compared using the Mann–Whitney U test. Categorical data were expressed as numbers and percentages and were compared by using the χ2 test or Fisher’s exact test. The correlation between CEUS features and surgical pathological results were compared by using univariate binary logistic regression. The clinical utility of US and CEUS were evaluated based on BI-RADS category 4A (4A, 4B, 4C, and 5 were defined as positive) and 4B (4B, 4C, and 5 were defined as positive) thresholds, respectively. The sensitivity, specificity, positive predictive value (PPV), and negative predictive value (NPV) were calculated. The sensitivity and specificity were compared by using the McNemar test. Statistical analyses were performed by using SPSS 25.0 (SPSS Inc). Differences were considered statistically significant at two-sided *P* values less than 0.05.

## Results

### Clinical data

**Fig. 1:**
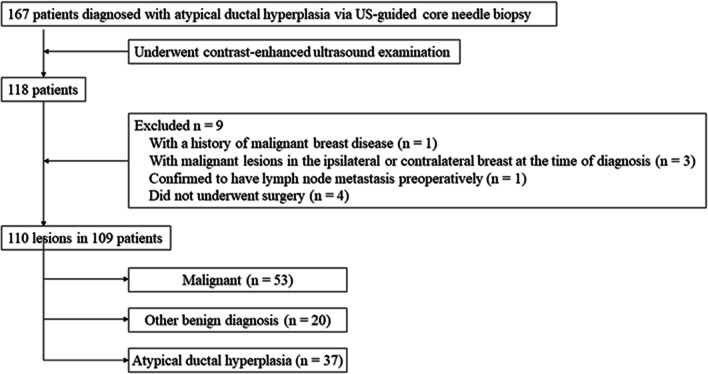
Flowchart of the process of patient enrollment

The mean age of the 109 women with 110 lesions was 49.7 years ± 11.6 (mean ± standard deviation), ranging from 28 to 76 years. No adverse events were reported during US, CEUS, and US-guided CNB procedures. According to the final surgical pathology results, 48.2% (53 of 110) of the 110 lesions were malignant, 33.6% (37 of 110) were ADH, and 18.2% (20 of 110) were other benign diagnosis (Fig. 1). Of the 53 malignant lesions, 69.8% (37 of 53) were DCIS and 30.2% (16 of 53) were invasive carcinoma. The median lesion size was 1.2 cm (interquartile range 0.8–2.1 cm; range 0.5–5.0 cm). Clinical data are summarized and compared in Table 1. The mean age and median lesion size were significantly higher in patients with upgrading than those of without upgrading (*P* < 0.001).

**Table 1:** Comparison of clinical data between patients with and without upgrade to malignancy after surgery

Variable	Entire cohort	Surgical pathology results		*P* value
		Malignant	Nonmalignant	
Number of lesions	110	53(48.2)	57(51.8)	
Mean age (year)^a^	49.7 ± 11.6	53.0 ± 12.1	44.8 ± 9.5	< 0.001
Median size (cm)^b^	1.2 (0.8–2.1)	1.7 (1.0–3.1)	0.9 (0.7–1.4)	< 0.001
Nipple discharge				0.215
Absent	94 (85.5)	43 (81.1)	51 (89.5)	
Present	16 (14.5)	10 (18.9)	6 (10.5)	
Palpability				0.213
Nonpalpable	31 (28.2)	12 (22.6)	19 (33.3)	
Palpable	79 (71.8)	41 (77.4)	38 (66.7)	
Biopsy needle				0.471
16-Gauge	48 (43.6)	25 (47.2)	23 (40.4)	
18-Gauge	6 2(56.4)	28 (52.8)	34 (59.6)	

Unless otherwise specified, variables are expressed as numbers of lesions with percentages in parentheses

^a^Variables are expressed as means with standard deviations in parentheses

^b^Variables are expressed as medians with interquartile ranges in parentheses

### CEUS findings

Univariate analysis of CEUS features showed that earlier wash-in time (OR, 16.93; 95% CI 2.14, 133.98; *P* = 0.007), hyper-enhancement (OR, 11.86; 95% CI 1.46, 96.40; *P* = 0.021), presence of perfusion defects (OR, 5.73; 95% CI 1.77, 18.52; *P* = 0.004), heterogeneous enhancement (OR, 5.51; 95% CI 2.13, 14.26; *P* < 0.001), ill-defined enhancement margin (OR, 7.95; 95% CI 3.31, 19.12; *P* < 0.001), irregular enhancement shape (OR, 8.38; 95% CI 3.42, 20.54; *P* < 0.001), enlarged size on CEUS (OR, 11.91; 95% CI 4.81, 29.50; *P* < 0.001) and presence of radial or penetrating vessels (OR, 33.94; 95% CI 4.35, 264.67; *P* = 0.001) were positively associated with malignant upgrading (Table 2). The CEUS score was 2 points in 15 lesions, 3 points in 33 lesions, 4 points in 42 lesions, and 5 points in 20 lesions.

**Table 2:** Comparison of contrast-enhanced ultrasound features between patients with and without upgrade to malignancy after surgery

Variable	Surgical pathology results		Univariable Analysis	
	Malignant	Nonmalignant	Odds Ratio (95%CI)	*P* value
*Wash-in time*				
Meantime/Later	1 (1.9)	14 (24.6)	1 (reference)	
Earlier	52 (98.1)	43 (75.4)	16.93 (2.14, 133.98)	0.007
*Enhancement degree*				
Hypo-enhancement	1 (1.9)	10(17.5)	1 (reference)	
Iso-enhancement	1 (1.9)	4 (7.0)	2.50 (0.12, 50.44)	0.550
Hyper-enhancement	51 (96.2)	43 (75.4)	11.86 (1.46, 96.40)	0.021
*Enhancement direction*				
Centrifugal/Diffuse	2 (3.8)	9 (15.8)	1 (reference)	
Centripetal	51(96.2)	48(84.2)	4.78 (0.98,23.26)	0.053
*Perfusion defects*				
Absent	37 (69.8)	53 (93.0)	1 (reference)	
Present	16 (30.2)	4 (7.0)	5.73 (1.77,18.52)	0.004
*Internal homogeneity*				
Homogeneous	7 (13.2)	26 (45.6)	1 (reference)	
Heterogeneous	46 (86.8)	31 (54.4)	5.51 (2.13,14.26)	< 0.001
*Enhancement margin*				
Well-defined	10 (18.9)	37 (64.9)	1 (reference)	
Ill-defined	43 (81.1)	20 (35.1)	7.95 (3.31,19.12)	< 0.001
*Enhancement shape*				
Round/Oval	9 (17.0)	36 (63.2)	1 (reference)	
Irregular	44 (83.0)	21 (36.8)	8.38 (3.42,20.54)	< 0.001
*Size on CEUS*				
Not enlarged	15 (28.3)	47 (82.5)	1 (reference)	
Enlarged	38 (71.7)	10 (17.5)	11.91 (4.81,29.50)	< 0.001
*Radial or penetrating vessels*				
Absent	33 (62.3)	56 (98.2)	1 (reference)	
Present	20 (37.7)	1 (1.8)	33.94 (4.35,264.67)	0.001

Variables are expressed as numbers of lesions with percentages in parentheses

**Fig. 2:**
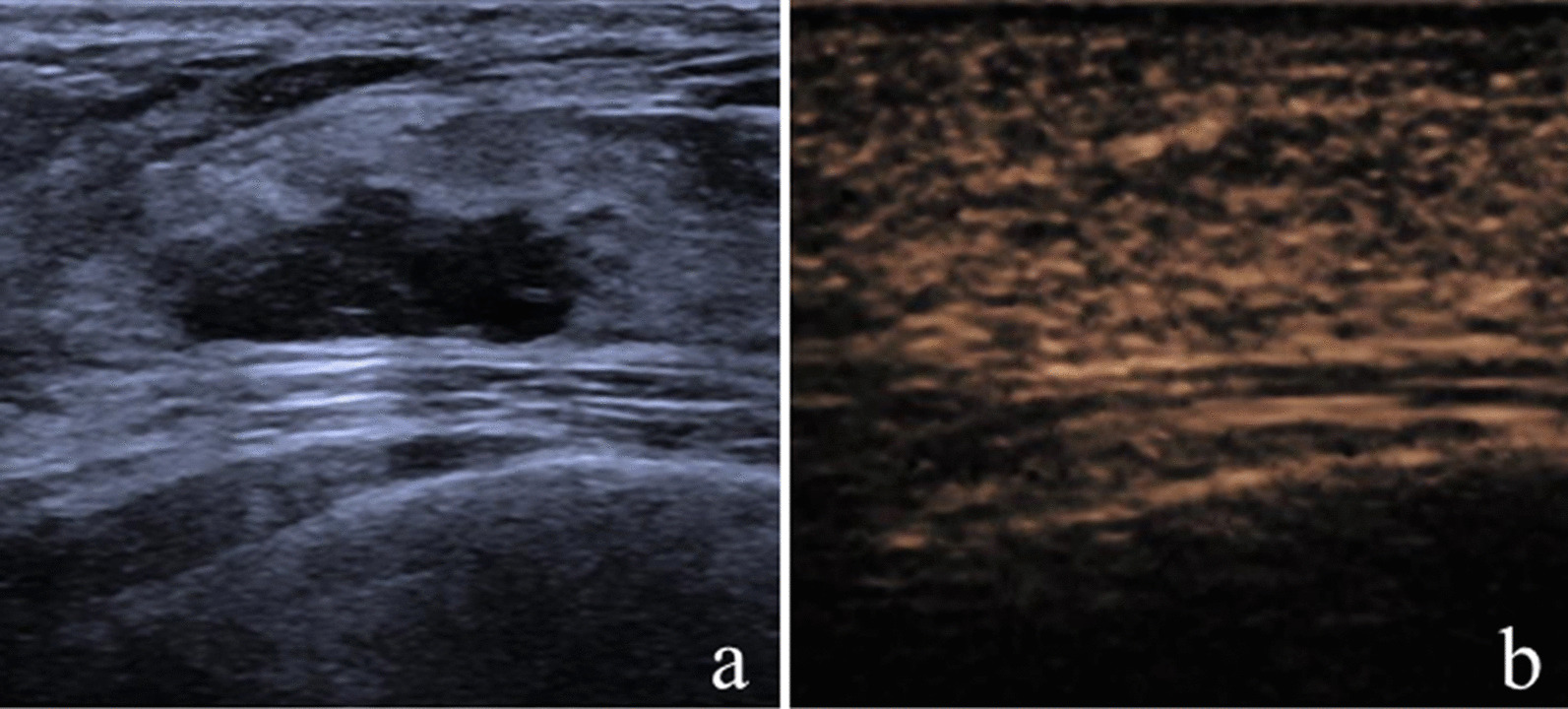
Image of a 30-year-old woman with a palpable mass. **a** B-mode ultrasound image;** b** contrast-enhanced ultrasound image showed that the lesion displayed iso-enhancement with the surrounding tissue. The CEUS score was 2 points. This lesion was originally classified as US BI-RADS 4A, downgraded to BI-RADS 3 after CEUS, and diagnosed as atypical ductal hyperplasia via US-guided core needle biopsy and as adenosis at surgery

**Fig. 3:**
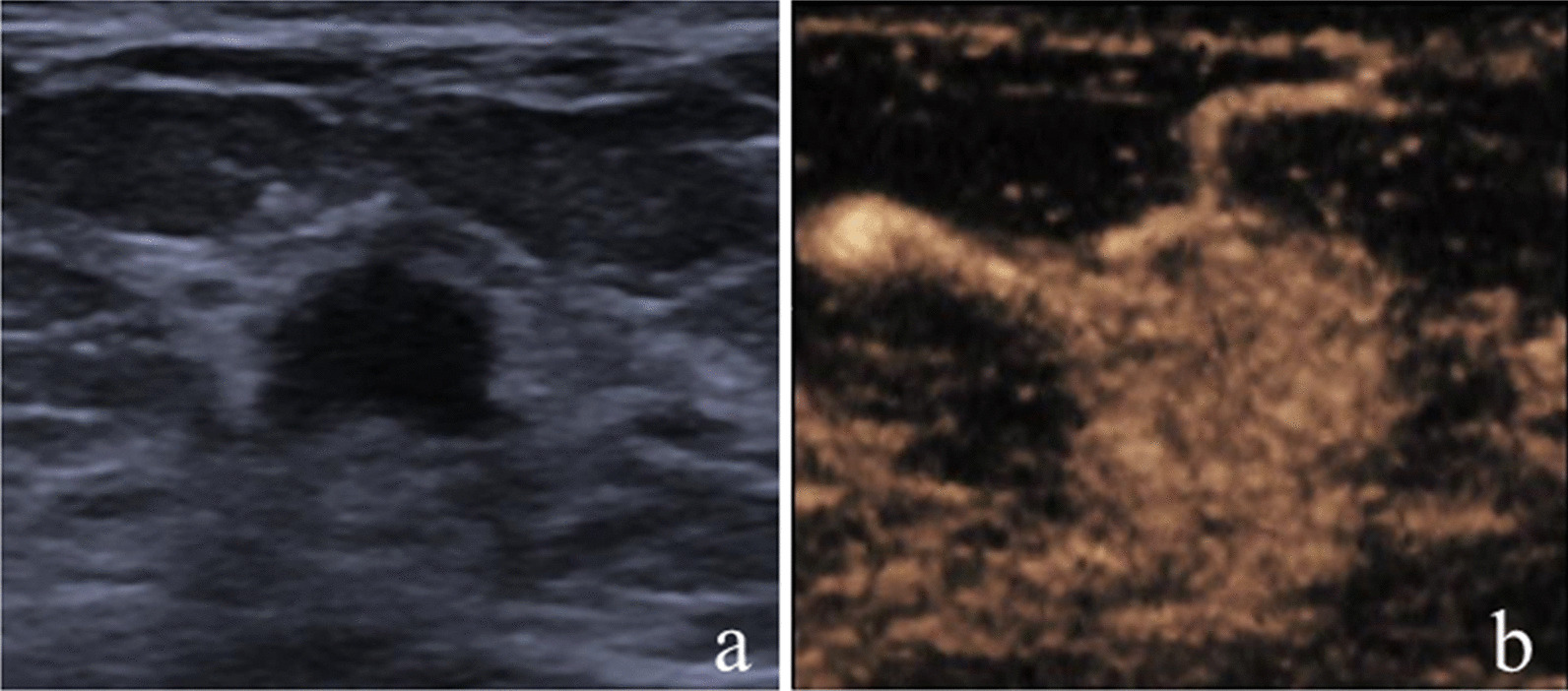
Image of a 42-year-old woman with a nonpalpable mass. **a** B-mode ultrasound image;** b** contrast-enhanced ultrasound image showed that the lesion displayed hyper-enhancement with ill-defined enhancement margin, enlarged size on CEUS, and penetrating vessels. The CEUS score was 5 points. This lesion was originally classified as US BI-RADS 4A, upgraded to BI-RADS 4B after CEUS, and diagnosed as atypical ductal hyperplasia via US-guided core needle biopsy and as ductal carcinoma in situ at surgery

**Fig. 4:**
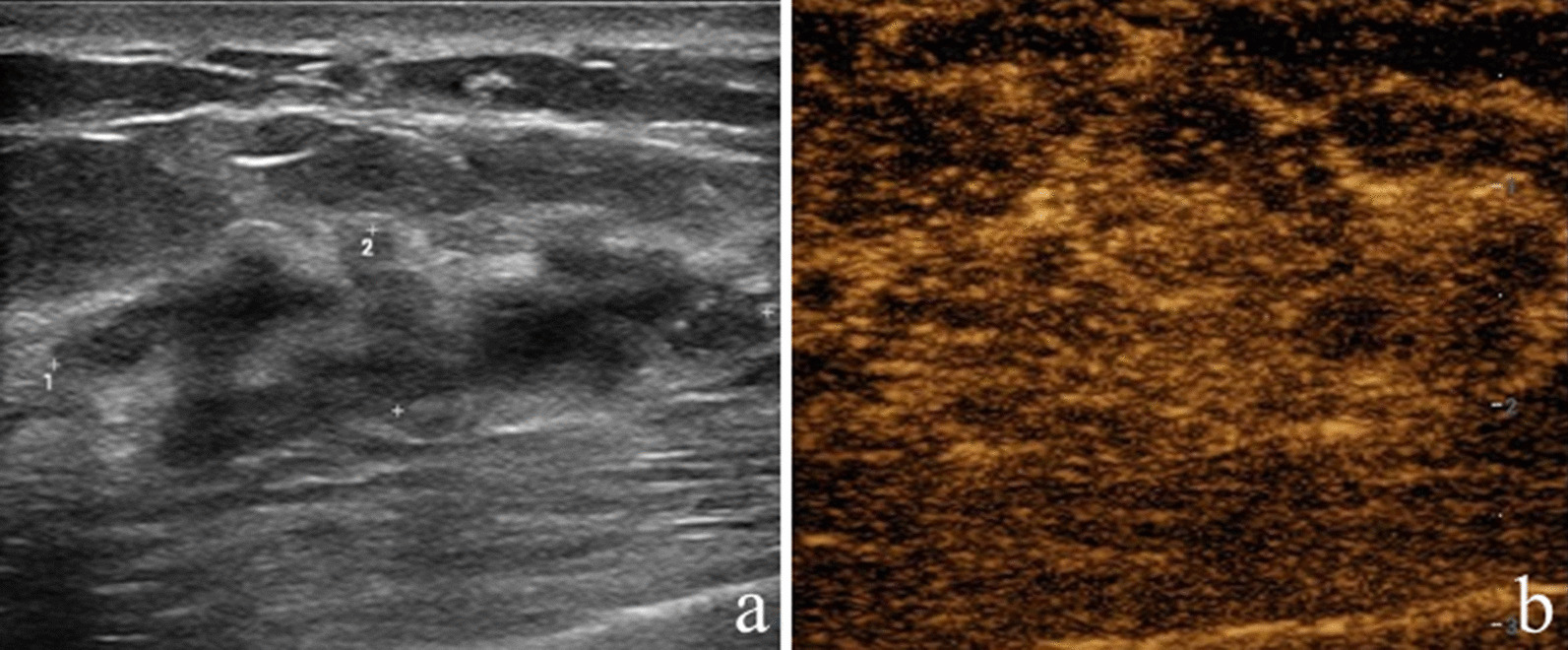
Image of a 60-year-old woman with a palpable mass. **a** B-mode ultrasound image;** b** contrast-enhanced ultrasound image showed that the lesion displayed iso-enhancement with the surrounding tissue. The CEUS score was 2 points. This lesion was originally classified as US BI-RADS 4B, downgraded to BI-RADS 4A after CEUS, and diagnosed as atypical ductal hyperplasia via US-guided core needle biopsy and as ductal carcinoma in situ at surgery

**Fig. 5:**
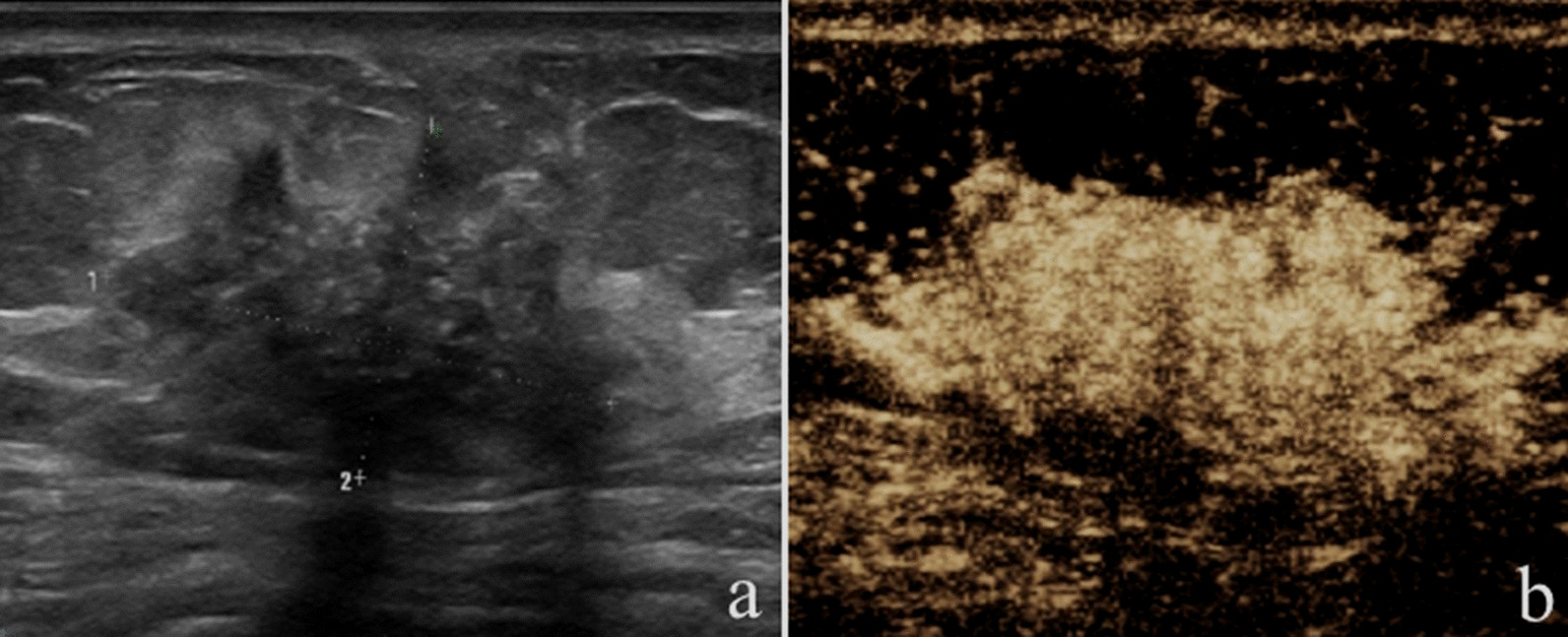
Image of a 38-year-old woman with a nonpalpable mass. **a** B-mode ultrasound image;** b** contrast-enhanced ultrasound image showed that the lesion displayed hyper-enhancement with ill-defined enhancement margin and enlarged size on CEUS. The CEUS score was 4 points. This lesion was originally classified as US BI-RADS 4C, upgraded to BI-RADS 5 after CEUS, and both were diagnosed as atypical ductal hyperplasia at US-guided core needle biopsy and at surgery

After incorporating CEUS into the original US BI-RADS, one malignant lesion and 13 nonmalignant lesions were reduced by one level; while, 43 malignant lesions and 14 nonmalignant lesions were increased by one level. Of the lesions with reduction in BI-RADS assessment, 92.9% (13 of 14) were nonmalignant, and of the lesions with increase in BI-RADS assessment, 75.4% (43 of 57) were malignant (Table 3; Figs. 2, 3, 4, 5).

**Table 3:** Changes of original US BI-RADS category after contrast-enhanced ultrasound

Original US BI-RADS	Reduce		No change		Increase	
	Malignant	Nonmalignant	Malignant	Nonmalignant	Malignant	Nonmalignant
3	0	0	0	6 (100)	0	2 (100)
4A	0	11 (100)	1 (5.6)	17 (94.4)	10 (62.5)	6 (37.5)
4B	1 (33.3)	2 (66.7)	2 (22.2)	7 (77.8)	24 (82.8)	5 (17.2)
4C	0	0	1 (100)	0	9 (90.0)	1 (10.0)
5	0	0	5 (100)	0	0	0
Total	1 (7.1)	13 (92.9)	9 (23.1)	30 (76.9)	43 (75.4)	14 (24.6)

Variables are expressed as numbers of lesions with percentages in parentheses

US, ultrasound; BI-RADS, Breast imaging reporting and data system

### Diagnostic performance of CEUS

When using a cutoff value of BI-RADS category 4A, the sensitivity, specificity, PPV, and NPV for US BI-RADS as compared with CEUS-adjusted BI-RADS were 100% versus 100%, 14.0% versus 29.8% (*P* = 0.022), 52% versus 57% (*P* = 0.481), and 100% versus 100%, respectively. After CEUS, 11 (all of them were nonmalignant) of the 45 US BI-RADS category 4A lesions were appropriately down-classified to BI-RADS 3, and 2 (both of them were nonmalignant) of the 8 original US BI-RADS category 3 lesions were inappropriately up-classified to BI-RADS 4A. Management decision making was not changed for the lesions with original US BI-RADS category 4B, 4C, and 5. At subgroup analysis of lesion size, the specificity of CEUS-adjusted BI-RADS for lesions ≤ 2 cm was significantly higher than that of original US BI-RADS (13.7% vs 31.4%, *P* = 0.012). For lesions > 2 cm, there was no significant difference in sensitivity, specificity, PPV, and NPV between CEUS-adjusted BI-RADS and original US BI-RADS. The diagnostic performance of US and CEUS based on the BI-RADS category 4A threshold was summarized and compared in Table 4.

**Table 4:** Diagnostic performance of ultrasound and contrast-enhanced ultrasound based on BI-RADS category 4A threshold

Variable	Sensitivity	Specificity	PPV	NPV
All				
US	100 (53/53)	14.0 (8/57)	52.0 (53/102)	100 (8/8)
US + CEUS	100 (53/53)	29.8 (17/57)	57.0 (53/93)	100 (17/17)
* P* value	–	0.022	0.481	–
> 2 cm				
US	100 (23/23)	16.7 (1/6)	82.1 (23/28)	100 (1/1)
US + CEUS	100 (23/23)	16.7 (1/6)	82.1 (23/28)	100 (1/1)
* P* value	–	> 0.99	> 0.99	–
≤ 2 cm				
US	100 (30/30)	13.7 (7/51)	40.5 (30/74)	100 (7/7)
US + CEUS	100 (30/30)	31.4 (16/51)	46.2 (30/65)	100 (16/16)
* P* value	–	0.012	0.505	–

Variables are expressed as percentages with numbers of lesions in parentheses; Breast Imaging Reporting and Data System (BI-RADS) category 4A, 4B, 4C, and 5 were defined as positive for calculation of diagnostic performance

US, ultrasound; CEUS, contrasted-enhanced ultrasound; NPV, negative predictive value; PPV, positive predictive value

When using cutoff value of BI-RADS category 4B, the sensitivity, specificity, PPV and NPV for original US BI-RADS as compared with CEUS-adjusted BI-RADS were 79.2% versus 96.2% (*P* = 0.012), 73.7% versus 66.7% (*P* = 0.289), 73.7% versus 72.9% (*P* = 0.917), and 79.2% versus 95.0% (*P* = 0.030), respectively. There were two cases of false negatives, one had both original US and CEUS-adjusted BI-RADS category 4A, and the other had an original US BI-RADS category 4B that was reduced to 4A after CEUS. Both of them were diagnosed as low-grade DCIS at finally surgical histology. After CEUS, 10 of the 45 original US BI-RADS category 4A lesions were appropriately up-classified to BI-RADS 4B; while, 6 of the 45 original US BI-RADS category 4A lesions were inappropriately up-classified to BI-RADS 4B. Additionally, 2 of the 41 original US BI-RADS category 4B lesions were appropriately down-classified to BI-RADS 4A; while, 1 of the 41 original US BI-RADS category 4B lesions was inappropriately down-classified to BI-RADS 4A. Management decision making was not changed for the lesions with original US BI-RADS categories 3, 4C, and 5. At subgroup analysis of lesion size, the sensitivity of CEUS-adjusted BI-RADS for lesions ≤ 2 cm was significantly higher than that of original US BI-RADS (76.7% vs 96.7%, *P* = 0.031). For lesions > 2 cm, there was no significant difference in sensitivity, specificity, PPV, and NPV between CEUS-adjusted BI-RADS and original US BI-RADS. The diagnostic performance of US and CEUS based on the BI-RADS category 4B threshold is summarized and compared in Table 5. For ADH assessment using CEUS-adjusted BI-RADS, the specificity of threshold 4B was significantly higher than that of threshold 4A (66.7% vs 29.8%, *P* < 0.001); while, there was no significant difference in sensitivity between threshold 4A and threshold 4B (96.2% vs 100%, *P* = 0.475).

**Table 5:** Diagnostic performance of ultrasound and contrast-enhanced ultrasound based on BI-RADS category 4B threshold

Variable	Sensitivity	Specificity	PPV	NPV
All				
US	79.2 (42/53)	73.7 (42/57)	73.7 (42/57)	79.2 (42/53)
US + CEUS	96.2 (51/53)	66.7 (38/57)	72.9 (51/70)	95.0 (38/40)
* P* value	0.012	0.289	0.917	0.030
> 2 cm				
US	82.6 (19/23)	83.3 (5/6)	95.0 (19/20)	55.6 (5/9)
US + CEUS	95.7 (22/23)	83.3 (5/6)	95.7 (22/23)	83.3 (5/6)
* P* value	0.375	> 0.99	0.919	0.580
≤ 2 cm				
US	76.7 (23/30)	72.5 (37/51)	62.2 (23/37)	84.1 (37/44)
US + CEUS	96.7 (29/30)	64.7 (33/51)	61.7 (29/47)	97.1 (33/34)
* P* value	0.031	0.289	0.966	0.135

Variables are expressed as percentages with numbers of lesions in parentheses; Breast Imaging Reporting and Data System (BI-RADS) category 4B, 4C, and 5 were defined as positive for calculation of diagnostic performance

US, ultrasound; CEUS, contrasted-enhanced ultrasound; NPV, negative predictive value; PPV, positive predictive value

## Discussion

Although current guidelines recommend open excision for all ADH lesions diagnosed by US-guided CNB, the management of ADH has been debated [2]. The essential problem is the inability to effectively identify lesions that are underestimated by CNB. This study showed that the combination of US and CEUS can effectively predict the malignant upgrading of ADH diagnosed by CNB, with a sensitivity, specificity, PPV, and NPV of 96.2%, 66.7%, 72.9%, and 95.0%, respectively, based on BI-RADS category 4B threshold.

Evaluating vascularity helps differentiate between benign and malignant lesions, as tumor growth requires extra vasculature to provide oxygen and nutrients [21]. Compared to color Doppler flow imaging, CEUS uses microbubbles to amplify vascular signals, improving the diagnostic performance of US [20]. Previous studies showed that the sensitivity and specificity of CEUS in breast lesion identification ranged from 91.9% to 95.5% and 75.6% to 88.7%, respectively [16, 19, 20, 22]. Our study indicated that the sensitivity of CEUS in predicting upgrade of ADH to malignancy resembled the sensitivity of CEUS reported in previous studies, but the specificity was inferior. Similar results were observed in a study of dynamic contrast-enhanced MRI, in which MRI achieved a high sensitivity of 94.1% and a low specificity of 60.7% for the identification of ADH upgrading [23]. ADH, a precancerous lesion with biological behavior similarities to low-grade DCIS [1, 24, 25], can be misdiagnosed as malignant by contrast-enhanced imaging, leading to false positives. Most false positive cases on MRI were eventually confirmed to be ADH after open excision [23]. Besides the CEUS and MRI, optoacoustic imaging in previous studies has also been used to evaluate the vascularity of tumors. Upgrading and downgrading the ultrasound BI-RADS categories of breast lesions using optoacoustic imaging can effectively improve the specificity without loss in sensitivity [26, 27]. However, the use of optoacoustic imaging in the evaluation of ADH diagnosed by CNB has not been reported. In the future, the combination of multiple imaging techniques may provide more useful information for individualized treatment strategies.

In this study, 95% of lesions classified to CEUS-adjusted BI-RADS category 3 and 4A were confirmed to be nonmalignant at surgery. Two false-negative cases were low-grade DCIS at surgery. According to the Second International Consensus Conference on lesions of uncertain malignant potential in the breast (B3 lesions), radiologic surveillance should not be recommended when underestimation rates exceed 10% for DCIS and 5% for invasive carcinoma [2]. This means that more than two-thirds of nonmalignant lesions (corresponding to a specificity of 66.7%) could be spared open excision in this study.

The subgroup analysis of the diagnostic performance of CEUS for different lesion sizes showed that CEUS performed better than the US in the diagnosis of lesions ≤ 2 cm, but not for lesions > 2 cm. The diagnostic performance of CEUS for breast was influenced by patients’ age and lesion size [20, 28, 29]. A multicenter study of CEUS for breast showed better diagnostic performance for lesions > 2 cm than for lesions ≤ 2 cm [28]. To obtain representative images, it is usually necessary to keep the plane selected for CEUS unchanged throughout the procedure. However, for larger CNB-diagnosed ADH lesions, the selected plane for CEUS may not correspond to the area of tumor cell distribution. This could explain the poor CEUS performance observed in lesions larger than 2 cm in this study. In this study, the sensitivity and NPV of lesions size > 2 cm were both numerically improved from 82.6% at US to 95.7% at CEUS and from 55.6% at US to 83.3% at CEUS. Despite these improvements, the numerical changes failed to carry statistical significance. Consequently, a large cohort study for lesions > 2 cm is necessary.

In this study, the management decision changes were observed exclusively in lesions with original US BI-RADS categories of 4A and 4B. The likelihood of malignancy corresponding to a CEUS score of 1 to 5 (0%, 5.6%, 8.1%, 81.5%, and 99.0%, respectively) [20] and the minimum acceptable limits (10% for DCIS and 5% for invasive carcinoma) [2] for surveillance were the main rationale for using CEUS to adjust original BI-RADS in this study. A previous study utilized CEUS to rerate BI-RADS by increasing or reducing one or two levels, achieving a specificity rate of 82.1% [20]. Nonetheless, reducing original BI-RADS by two levels may improve specificity but simultaneously decrease sensitivity, which may not be favorable for the evaluation of ADH in CNB. In our cohort, 92.9% of the lesions with BI-RADS reduction were nonmalignant, and 75.4% of lesions with an increase in BI-RADS were malignant. Based on threshold 4B, management decisions did not changed for lesions with BI-RADS 3, 4A, and 5 after CEUS, suggesting that CEUS does not provide value for these types of lesions.

There are some limitations in our study. First, this study was a single-center study with a small sample size. Some potentially valuable data such as sensitivity and NPV of lesions > 2 cm did not translate into statistical significance. Second, only the patients who underwent the US, CEUS, and US-guided CNB were included in the analysis, which may result in selection bias. Third, there was an absence of prospective validation and external validation with data from other institutions.

## Conclusions

CEUS is helpful in predicting malignant upgrading of ADH, especially for lesions smaller than 2 cm and those classified as BI-RADS 4A and 4B on ultrasound. Further studies with larger sample sizes are necessary to confirm the clinical applicability of this finding.

## Data Availability

The data from the patients’ database of Chinese PLA General Hospital are not publicly available as it contains sensitive information. To access the data, a request for extraction must be made to the Chinese PLA General Hospital. The institution requires an ethical approval to access the data.

## Abbreviations

ADH: Atypical ductal hyperplasia
DCIS: Ductal carcinoma in situ
CNB: Core needle biopsy
CEUS: Contrast-enhanced ultrasound
OR: Odds ratio
